# Cuff overinflation and endotracheal tube obstruction: case report and experimental study

**DOI:** 10.1186/1757-7241-18-18

**Published:** 2010-04-08

**Authors:** Christian Hofstetter, Bertram Scheller, Sandra Hoegl, Martin G Mack, Bernhard Zwissler, Christian Byhahn

**Affiliations:** 1 Clinic of Anesthesiology, Intensive Care Medicine, and Pain Therapy, J.W. Goethe-University Hospital Frankfurt, Germany; 2 Institute of Anesthesiology and Critical Care Medicine, University of Mannheim, Germany; 3 Department of Anesthesiology, Ludwig Maximilians University of Munich, Germany; 4 Department of Diagnostic and Interventional Radiology, J.W. Goethe-University Hospital Frankfurt, Germany

## Abstract

**Background:**

Initiated by a clinical case of critical endotracheal tube (ETT) obstruction, we aimed to determine factors that potentially contribute to the development of endotracheal tube obstruction by its inflated cuff. Prehospital climate and storage conditions were simulated.

**Methods:**

Five different disposable ETTs (6.0, 7.0, and 8.0 mm inner diameter) were exposed to ambient outside temperature for 13 months. In addition, every second of these tubes was mechanically stressed by clamping its cuffed end between the covers of a metal emergency case for 10 min. Then, all tubes were heated up to normal body temperature, placed within the cock of a syringe, followed by stepwise inflation of their cuffs to pressures of 3 kPa and ≥12 kPa, respectively. The inner lumen of the ETT was checked with the naked eye for any obstruction caused by the external cuff pressure.

**Results:**

Neither in tubes that were exposed to ambient temperature (range: -12°C to +44°C) nor in those that were also clamped, visible obstruction by inflated cuffs was detected at any of the two cuff pressure levels.

**Conclusions:**

We could not demonstrate a critical obstruction of an ETT by its inflated cuff, neither when the cuff was over-inflated to a pressure of 12 kPa or higher, nor in ETTs that had been exposed to unfavorable storage conditions and significant mechanical stress.

## Introduction and Case

Frequent causes for critical obstruction of a cuffed ETT include kinking, secretions and cuff hernia [1,2]. This study was initiated by the observation of a case of critical endotracheal tube (ETT) obstruction due to a compression of its confining wall by the inflated cuff.

**Figure 1:**
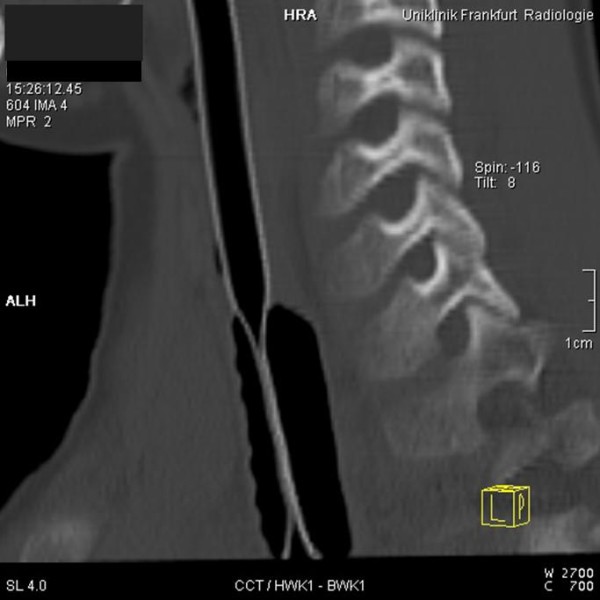
**Computed tomography showing the sagittal plane of cervical spine and trachea**. The cuff compresses the inner lumen of the endotracheal tube, thus leading to critical obstruction.

**Figure 2:**
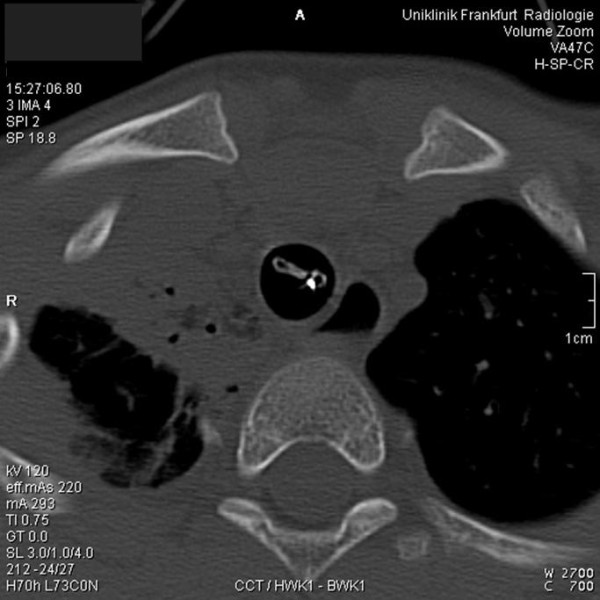
**Computed tomography**. Transversal plane at the level of the first thoracic vertebra. The endotracheal tube is critically obstructed by the inflated cuff. The radiopaque label (white dot on the scan) indicates the obstructed lumen of the tracheal tube.

An eight year old boy was admitted to the emergency room of our institution, suffering from multiple injuries caused by a traffic accident. Tracheal intubation with a cuffed 6.0 mm internal diameter (ID) ETT - the manufacturer of which could not be determined - was performed at the site of the accident, and ventilation was so far uneventful. According to our institutional trauma management protocol, a whole body computed tomography (CT) scan was performed. There was no reason to assume a pneumothorax. Due to increasing inspiratory airway pressures (>4 kPa) accompanied by arterial hypotension the CT scan was prematurely aborted. Manual ventilation affirmed high inspiratory airway resistance, and auscultation showed the absence of breath sounds over both lungs. Advancing a suction catheter through the ETT was not possible, neither could any material potentially causing the obstruction be aspirated. Therefore, the ETT was immediately removed under direct laryngoscopy and the boy's trachea reintubated with another cuffed ID 6.0 mm ETT (Lo-Contour Magill, Mallinckrodt, Athlone, Ireland). A cuff pressure of 2 kPa was measured with a cuff manometer (Mallinckrodt Cuffmanometer, Mallinckrodt, Athlone, Ireland). Immediately after re-intubation, ventilation parameters returned to normal. Subsequent review of the previously obtained CT scan data revealed the cause of the ETT obstruction. As shown in Figures 1 and 2, the inflated cuff of the ETT compressed its confining wall and critically obstructed its lumen. As a consequence, high inspiratory airway pressures must have resulted from a critically occluded ETT-lumen.

An almost complete obstruction of the cross-sectional area of an ETT by its cuff has not been reported in the literature yet. Two potential reasons could have caused this life threatening complication: Faults of the material itself or improper finishing of the ETT could have been one aspect, damage of the material due to suboptimal storage in the ambulance car another.

Because a definitive clarification of the causative reason for this serious complication was not possible posthoc, we performed a prospective study. To clarify whether different commercially available standard ETTs expose such a problem, we stored two of each kind for one year at simulated conditions comparable to those in an ambulance car. In addition, one tube of each kind was improperly handled by clamping it between the top covers of an emergency case for 10 min.

## Materials and methods

We asked five manufacturers of endotracheal tubes to send us at least three ETT of the sizes 6.0, 7.0 and 8.0 mm ID for a prospective ex vivo study. No details concerning the intended project were communicated. The following types of cuffed ETT were exposed to the conditions described below: Rueschelit Super Safety Clear (Ruesch, Kernen, Germany); Vygon 518 (Vygon, Ecquen, France); ASID Bonz Endosoft-Plus, ASID Bonz, Boeblingen, Germany); Lo-Contour Magill (Mallinckrodt, Athlone, Ireland); Medisil Murphy (Hudson, Lohmar, Germany). Two originally wrapped ETTs of each manufacturer were deposited within a commercially available aluminum emergency case (Ulmer Koffer I, Weinmann, Hamburg, Germany) for 13 months. The case was placed in ambient environment outside a building unprotected against sun and wind for the entire study period and thus exposed to temperatures ranging from -12°C to +44°C. Temperatures inside the case were continuously recorded with a digital thermometer (Vega, WML, Haren, Germany). The case was not opened or moved within these 13 months.

After completion of the storage protocol, one ETT of each manufacturer was unwrapped and heated for 30 min in a water bath with a temperature of 37°-38°C. After the heating period the ETT was immediately positioned with its cuffed end into the cock of a plastic syringe serving as a model for the trachea, a method that has been used by other authors before [3,4]. ETTs with an ID of 6.0 mm were put into a 10 ml syringe, and ETTs with an ID of 7.0 and 8.0 mm into a 20 ml syringe (Discardit II, Becton Dickinson, Fraga, Spain), respectively. Before use, the syringes were also heated in the water bath for 30 min. Then, the cuff of the ETT was inflated with a cuff manometer (Mallinckrodt Cuffmanometer, Mallinckrodt, Athlone, Ireland) up to a pressure of 3 kPa for 10 min. The inner lumen of the ETT was checked with the naked eye for any obstruction caused by the external cuff pressure.

The second wrapped tube of each manufacturer was clamped between the two top covers of an emergency case (Ulmer Koffer I, Weinmann, Hamburg, Germany) with its cuffed end, including the whole length of the cuff in its midline by completely closing the case for 10 min. Thereafter, each ETT was heated to a temperature of 37°-38°C as described above. These ETTs were also introduced into the cock of a heated 10 or 20 ml syringe, the cuff inflated, and the tube's lumen checked for obstruction.

When no sign of tube obstruction was observed at a cuff pressure of 3 kPa, the cuff was further inflated with a total volume of 10 ml of air by using a syringe. Subsequently, the pilot tube was branched off with a plastic clamp and the cuff pressure manometer was connected to detect the actual cuff pressure.

## Results

None of the tracheal tubes that were stored in the emergency case for more than one year under ambient outside conditions showed any visible obstruction of its inner lumen when the cuff was inflated to 3 kPa. Further inflation of the cuff with a total volume of 10 ml of air resulted in cuff pressure exceeding 12kPa (upper detection limit of the manometer used) in all cases. Even the application of that excessive pressure did not result in visible obstruction of any of the tubes studied. Likewise, no visible obstruction could be generated by using the same protocol in any of those tubes that were previously clamped between the covers of the emergency case.

## Discussion

Based on a clinical case in which a tracheal tube was obstructed by external pressure from its inflated cuff for unknown reasons, we aimed to determine factors potentially supporting such tube obstruction in an ex vivo study. Different disposable endotracheal tubes were therefore exposed to extreme conditions of temperature and mechanical stress. Although such conditions are unlikely to occur in the hospital environment, they may be observed in prehospital settings, e.g. in ambulance cars that are exposed to ambient climate around the year. We were, however, not able to reproduce any visible tube obstruction.

When using cuffed endotracheal tubes, cuff pressure monitoring is strongly recommended to avoid hyperinflation and, mostly feared, subsequent tracheal mucosal damage [5]. Therefore, cuff pressures of 3.3kPa are recommended not to be exceeded. However, in the underlying clinical case the cuff pressure has not been determined, neither by the physicians on the scene nor on hospital admission.

It remains speculative when the critical obstruction of the ETT occurred. In the prehospital setting, endotracheal intubation is usually performed under pressure of time in emergency situations. Therefore, rapid and uncritical inflation of an ETT cuff by an air bolus (e.g. 10ml) may result in inadequately high cuff pressure often exceeding 4kPa [6]. Therefore, we decided to inflate the cuff with 10ml of air to simulate ordinary out-of-hospital customs even if the initially applied cuff pressure of 3kPa did not result in ETT obstruction.

In the underlying case, various factors could have resulted in the observed acute increase of airway pressure, such as insufficient depth of anesthesia, tension pneumothorax, tube dislodgement, obstruction by secretions or kinking of the tube or breathing circuit. However, all such potential reasons were quickly ruled out. Facing the problem persisting, the responsible anesthesiologist decided to remove the tracheal tube and to re-intubate the boy's trachea with a new ETT of the same size. The removed ETT that caused the problem did not look conspicuous after extubation and therefore was discarded. Unfortunately, when the CT scan identified the tube's cuff as the cause of the problem, the waste - including this tube - was already removed from the emergency room and could not be located anymore. The examination of this ETT would have been of special impact for the clarification of the complication since deficiencies of the material might have been responsible for the complication.

The respective ETT was part of the equipment of an ambulance car and stored in an aluminum emergency case for a certain period of time which, however, retrospectively could not exactly be identified. According to the information from the emergency physician it was highly likely that this tube has been stored in the emergency case inside the ambulance car for months before it was used. This is absolutely possible since prehospital tracheal intubation in children is rare and thus ETT sizes of 6.0mm ID and smaller are seldom used. Therefore it is likely that the tube was stored under suboptimal conditions and may have been exposed to extreme variations of temperature and climate for a considerable period of time. Moreover, since space in emergency cases is limited, tubes are often stored in a very compact manner including external pressure from other solid equipment. Even clamping between the covers of these cases may occur. As reported by the emergency physician, the package of the ETT was intact and a short test showed leak tightness (cuff inflation with 5-10ml of air for 10 sec.) immediately prior to its use.

Stuart and co-workers in 1994 reported on a series of ETT obstruction caused by over-inflated cuffs resulting in cuff herniation and compression of the soft distal portion of a wire reinforced silicone tube [4]. However, in this report, the mechanism leading to critical obstruction of the ETT was different to that in our clinical case. On the one hand, the authors reported on a wire reinforced silicone ETT with a soft tip that obviously could be compressed very easily. Further, this observation could be reproduced, specifically in ETTs that had been autoclaved several times [4].

The tracheal models used in the present study consisted of a rigid polyvinylchloride (PVC) tube with internal diameters of 15mm in case of the 10 ml syringe and 20mm when a 20 ml syringe was used, respectively. These diameters correspond well to the age-related internal tracheal diameters of patients for whom ID 6.0-8.0mm cuffed ETT are recommended [7].

The use of a rigid model, however, does not reflect tracheal wall compliance *in vivo*. It has been shown that tracheal wall compliance is different in the anterior, posterior or lateral part of the trachea [8]. Nevertheless, we believe that the use of a rigid trachea-model is a stronger approach to clarify the question of the present study since a rigid, non-compliant PVC tube transmits the entire pressure of its cuff to its wall.

In conclusion, we could not reproduce the event of a critical obstruction of an ETT by its inflated cuff, neither when the cuff was overinflated to >12 kPa, nor in tubes that had been exposed to unfavorable storage conditions and significant mechanical stress. However, the sample size was too small to extrapolate these results into a general recommendation. We would therefore be very pleased if these results would lead to a manufacturer-driven trial with a sufficient sample size.

## Competing interests

The authors declare that they have no competing interests.

## Authors' contributions

CH has made substantial contributions to conception, acquisition of data and drafting the article. BS and SH have made substantial contributions to analysis, interpretation of data and in drafting the article. MGM has made substantial contributions to analysis and interpretation of data. BZ has made substantial contributions to conception and revised the manuscript critically for important intellectual content. CB has made substantial contributions to conception, acquisition of data and revised the manuscript. All authors read and approved the manuscript.
